# Harm Perception in Response to Pictorial Warning Labels Predict Higher Non-Smoking Intention among Korean Adolescents

**DOI:** 10.3390/ijerph18041404

**Published:** 2021-02-03

**Authors:** Jun Hyun Hwang, Soon-Woo Park

**Affiliations:** Department of Preventive Medicine, Catholic University of Daegu School of Medicine, Daegu 42472, Korea; pmdr213@cu.ac.kr

**Keywords:** pictorial warning label, harm perception, non-smoking intention, tobacco control

## Abstract

Because of recent controversy surrounding the use of excessively graphic pictorial warning labels (PWL) on cigarette packs in South Korea, it is necessary to provide evidence to evaluate their effectiveness as a tobacco control policy according to the harm perception they evoke. An analysis was performed using a nationally representative survey conducted six months after the introduction of PWLs in South Korea. Among 62,276 survey participants, 43,231 students from grades 7–12 who had seen a PWL in the past 30 days comprised the research sample. Non-smoking intention was evaluated according to the harm perception evoked by PWLs, which predicted higher non-smoking intention among adolescents. Non-smoking intention was particularly higher in daily smokers with harm perception (52.0%) than those who have never smoked and have no harm perception (40.1%). In the case of those who perceived harmfulness, non-smoking intention was formed in about 45% of daily smokers who had no experience of trying to quit in the last 12 months. Therefore, it is important to select PWLs that can arouse in adolescents sufficient harm perception of smoking.

## 1. Introduction

The well-designed health warning messages on cigarette packs are a key component in comprehensive tobacco control policies that serve as cost-effective measures to raise public awareness on smoking and reduce tobacco use [1]. The use of a pictorial warning label (PWL) is considered more effective than a text-only warning [2]. It was introduced in Canada in 2001 and has since spread worldwide to 100 countries and jurisdictions as of 2016 [3]. The World Health Organization (WHO) Framework Convention on Tobacco Control (FCTC) requires that warning labels including the PWL take up more than 50% of the cigarette pack’s primary display area [1,4].

In several countries, the introduction of the PWL has been delayed for similar reasons. In the case of the United States, tobacco companies argued that the PWL proposed by the Food and Drug Administration (FDA) in 2011 was unduly burdensome and violated the First Amendment, and D.C. Circuit held the FDA rules in 2012 [5]. In Korea, the introduction of the PWL was delayed because it violated the rights of tobacco companies and threatened smokers’ rights of happiness [6]. Over 10 years after Korea first made efforts to pursue the bill on PWLs in 2002, it was finally enacted in June 2015 [7]. There was some opposition to the introduction of warning pictures in Korea, prompting the following legal provision to be added [8]: “That the warning pictures shall be based on facts without being overly obnoxious.”

According to the Message Impact framework, which proposes the mechanism of the effect of PWLs based on communication and psychological theory, PWLs sequentially affect attention and recall, warning reactions, attitudes and beliefs, intentions, and ultimately, behavior [9]. In a clinical trial on the influencing mechanism of PWLs on smokers’ quit attempts, PWLs were proven effective through mediation effects on attention, negative effects, social interactions, and thoughts on warnings and the harmful effects of smoking [10]. Other experimental studies supported the differential effects of PWLs according to the level of emotional reactions. In two experimental studies on smokers in the United States, a PWL causing higher negative emotions induced a higher quit intention among smokers [11,12]. 

Since most of the intermediate factors used in the fore mentioned studies were evaluated using subjective survey items, the effect of PWLs may vary depending on the individual’s perception of it. One of the main reasons for the opposition against the introduction of the PWL—that it is unduly burdensome or overly obnoxious—is difficult to objectively evaluate, which challenges the method to increase the PWL’s effectiveness. As in the United States and Korea, there is a possibility that the controversy surrounding the selection of PWLs may arise any time in other countries preparing to introduce it or when it comes time to replace them. As a result, it is possible to delay its introduction or even weaken its effects after. As such, subjective perception after exposure to PWL can either act as an obstacle in its introduction or play an important role in maximizing its effect, making the collection of sufficient scientific evidence on it essential. Since the aforementioned studies were only conducted on smokers [11,12], it is necessary to evaluate the effects of PWLs on non-smokers. After the introduction of the PWL in Korea, studies on its effects were conducted, but differences in the effect of individuals’ perception by exposure to PWL were not evaluated [13,14]. 

Our study aims to evaluate the relationship between the level of the harm perception evoked by PWL and non-smoking intention in the general population that includes non-smokers. For this purpose, we hypothesize that the harm perception in response to PWLs predicts a higher non-smoking intention in the general population of Korean adolescents. 

## 2. Materials and Methods

### 2.1. Study Sample

Data from the 13th Korea Youth Risk Behavior Web-Based Survey (KYRBS-XIII) conducted in 2017 by the Korea Centers for Disease Control and Prevention (KCDC) were analyzed for this study. The KYRBS is a nationally representative, self-reported, and anonymous online survey administered to Korean students enrolled in grades 7 to 12 (middle and high school grades). It uses a stratified multistage probability sampling design to produce nationally representative statistics on health behaviors of Korean adolescents. A total of 62,276 students from 799 schools (399 middle schools and 400 high schools) completed the KYRBS-XIII (response rate = 95.8%). Of all participants, the study population included those whose responses indicated that they had seen a PWL in the past 30 days (N = 43,231, 69.4% of all KYRBS-XIII participants). Participants who were exposed to PWLs were then asked to answer an additional questionnaire to assess their warning reactions to them. Additional details about the sampling method and survey procedure are available elsewhere [15]. The KYRBS was approved by the Institutional Review Board of the KCDC (2014-06EXP-02-P-A). 

### 2.2. Measures

For those who were exposed to PWLs, two perceived warning reactions (harm perception, non-smoking intention) were evaluated using the following questions, respectively: (1) “To what extent, if at all, do the warning labels on cigarette packs make you think about the health risks of smoking?”, (2) “To what extent, if at all, do the warning labels on cigarette packs make you think about the intention not to smoke?” The possible responses to these questions were: (1) “not at all,” (2) “a little,” (3) “somewhat,” and (4) “a lot.” These responses were reclassified into two categories: “yes” for (3) somewhat and (4) a lot; and “no” for (1) not at all and (2) a little.

Other covariates were categorized into two domains: sociodemographic and smoking-related factors. Sociodemographic variables included sex, grade (7–12), residential area (metropolitan, small- or medium-sized city, or rural), perceived household economic status (high, middle, or low), and perceived academic performance (high, middle, or low). Smoking-related factors included participation in smoking prevention education during the last 12 months (yes or no), exposure to anti-tobacco messages in the last 12 months (yes or no), and smoking status (never a smoker, former smoker, and current smoker). Current smoker was further reclassified into three groups: infrequent smoker (1~19 days per month), frequent smoker (20~29 days per month), or daily smoker by frequency of smoking [16].

### 2.3. Statistical Analysis

The weighted percentages of the warning reactions (harm perception of smoking, non-smoking intention) were calculated. A logistic regression was used to evaluate the factors associated with, and the relationship between, the harm perception of smoking and non-smoking intention. In the analysis of factors related to non-smoking intention, two models were applied depending on whether harm perception of smoking was included. To evaluate the differential effects of harm perception by smoking status, the odds ratio (OR) for non-smoking intention was calculated according to the combination of harm perception and smoking status. In addition, non-smoking intention in current smokers can be influenced by their attempts to quit smoking. Therefore, to exclude the effect of quit attempts, a subgroup analysis was conducted on current smokers to examine the effect of harm perception on their non-smoking intention after they were stratified based on experience of trying to quit in the last 12 months. All analyses were performed using the SPSS version 19.0 (IBM, Armonk, NY, USA). A *p*-value < 0.05 indicates statistical significance. Complex SPSS sampling methods were used to accurately represent Korean adolescents.

## 3. Results

The characteristics of the study participants and their warning reactions to PWLs are shown in Table 1. Of the total participants exposed to PWLs, 83.9% and 83.1% had harm perceptions of smoking and non-smoking intention, respectively. The formation of harm perception and non-smoking intention after exposure to PWLs was higher in females, students in the lower grades, and those who participated in smoking prevention education or were exposed to anti-tobacco messages. The maximum gap in harm perception and non-smoking intention according to smoking status were about 45%p and 64.7%p, respectively, showing a clear dose–response relationship. The formation of non-smoking intention was higher in those who perceived the harmfulness of smoking after exposure to PWLs (93.4%), than those who did not (29.5%).

Table 2 illustrates the factors related to the warning reactions after exposure to PWLs. The logistic regression analysis revealed that females, students from lower grades, those with middle and low perceived academic performance, and those who participated in smoking prevention education or were exposed to anti-tobacco messages, were more likely to perceive the harmfulness of smoking after exposure to PWLs. Similarly, the formation of non-smoking intention after exposure to PWL was also higher in females, students from lower grades, and those who participated in smoking prevention education or exposed to anti-tobacco message. The effect of PWL exposure on warning reactions (harm perception, non-smoking intention) was particularly prominent according to the smoking status with an adjusted OR of 7.10 (95% confidence interval [CI] 6.33–7.96) and 18.43 (95% CI 16.34–20.79 in Model 1), respectively. In Model 2, which contains the factor of harm perception, harm perception of smoking had the highest adjusted OR value of 28.38 (95% CI 26.37–30.53) for non-smoking intention, and the intensity of the relevance of other factors including smoking status became weaker (Table 2).

In each group classified by harm perception of smoking, non-smoking intention was the lowest in daily smokers, and the highest in those who have never smoked, showing a clear dose–response relationship by smoking status (group without harm perception: 3.7% to 15.24%, group with harm perception: 52.0% to 95.7%). Considering both harm perception and smoking status at the same time, non-smoking intention was higher in the group with harm perception than in the group without; non-smoking intention was higher in daily smokers with harm perception (52.0%), than those who have never smoked without harm perception (40.1%). The adjusted OR for non-smoking intention increased significantly with the harm perception of smoking and smoking status (adjusted OR range compared to daily smokers without harm perception: 1.99 [95% CI 1.10–3.59] to 466.11 [95% CI 331.75–654.88], p for trend < 0.001), Particularly, the adjusted OR increased sharply among those who perceived the harmfulness of smoking in the same level of smoking status (Table 3). 

Table 4 shows the results of analyzing current smokers’ non-smoking intention by stratifying the experience of trying to quit smoking during the last 12 months. As with the results including non-smokers in Table 3, in both groups stratified by their experience of trying to quit smoking, the adjusted OR increased according to the combination of harm perception and level of smoking status, having the highest adjusted OR value for non-smoking intention in those who have never smoked with a harm perception of smoking. Overall, the non-smoking intention was higher in the group that had an experience of trying to quit smoking (4.7% to 69.0%) than in the group that did not (1.7% to 58.8%), but the increase in adjusted OR value by harm perception after exposure to PWL was steeper in the latter (adjusted OR range compared to daily smoker without harm perception: 4.12 [95% CI 1.12–15.18] to 90.70 [95% CI 42.19–195.00] vs. 1.58 [95% CI 0.81–3.07] to 46.19 [95% CI 29.82–71.56] in the former group) (Table 4).

## 4. Discussion 

The effect of PWLs on the formation of non-smoking intention may vary depending on various factors. Among them, harm perception evoked by PWLs has the greatest effect on non-smoking intention among Korean adolescents. This study showed results that are in line with those of previous experimental studies on smokers that indicated how the effect of PWLs was stronger when they evoked more negative emotions [11,12]. Several studies explained this association as a mediation effect of the negative affect evoked by PWLs [9,10,17]. 

This study went one step further from previous studies and evaluated the effect of harm perception on non-smoking intention according to smoking status. Focusing on the two strongest predictors of non-smoking intention, harm perception evoked by PWLs and smoking status, we identified the possibility that the level of perceived harmfulness of smoking evoked by PWLs could be one of the strategies to maximize its effect. In general, the effect of a PWL is known to be greater in non-smokers than current smokers [9], but we suggest that it can be more effective in smokers than non-smokers according to the perceived harmfulness of smoking it evokes. Additionally, when only evaluating the effect of a PWL on smokers, its harm perception had a great influence on the formation of non-smoking intentions, even in those who had not experienced trying to quit smoking in the last 12 months. 

Based on the results of this study, selecting PWLs that raise arousal has the following expected effects. First, a universal effect can be expected among smokers regardless of smoking status or experience of trying to quit. The result of a meta-analysis showed that the effect of PWLs was lower in smokers [9], and an experimental study suggested that PWLs had no significant expected effect in regular adolescent smokers [18]. However, considering that non-smoking intention was formed in about 45% of daily smokers who have not tried to quit, our study supports that PWLs, designed so that the harmfulness of smoking is fully recognizable, may have a universal impact on the majority of people. Second, selecting PWLs that raise arousal can induce a lasting effect because when the effects of a PWL evoke a high emotional response, they can last a long time [19,20]. The WHO FCTC recommends the periodic rotation of warning labels as a measure against a wear-out effect over time. However, the rotational period may differ depending on the political situation of implementing the use of PWLs because the WHO’s recommended rotation cycle is every 12 to 36 months [1]. Therefore, selecting PWLs that evoke a high emotional response can be a supplemental method to sustain the effect of PWLs for a set period of time. 

This research has policy implications for tobacco regulations related to PWLs, and provides evidence which necessitates raising their arousal level. The introduction of PWLs is being opposed because of their inappropriateness, which is unduly burdensome or overly obnoxious. In Korea, these restrictive phrases have been specified in the enactment of the law [8], and the government’s initial press release stated that PWLs used in Korea were less graphic than foreign PWLs [21]. However, as shown in this study, since the perceived harmfulness of smoking evoked by PWLs plays a key role in increasing its effectiveness, it is possible to justify selecting PWL types that can maximize its effect through the research results. Moreover, research results show that PWLs that induce low negative emotions are less effective than text-only warning labels [12]. Therefore, it is necessary to strengthen the warning level of PWLs. 

Despite low smoking prevalence in adolescents, a total of about 70% of Korean adolescents were exposed to PWLs [14], and non-smoking intention was formed in about 80% of them. Adolescents serve as the marketing targets of tobacco companies [22] and are exposed to tobacco advertisements from an early age. Their exposure to these advertisements affects their smoking initiation [23], but strengthening the use of PWLs in terms of harmfulness could lower their intention to start smoking. This association was also supported by a longitudinal study that indicated how negative images on cigarette packs lower the youth’s intention to smoke [24]. In this study, the harm perception rate by PWL was higher in students in lower grades. This perceived harmfulness of smoking from an early age leads to the formation of non-smoking intentions, which are attributed to social norms for smoking in adolescents, and can ultimately affect the decrease in smoking prevalence.

The result of this study should be interpreted with caution due to the following limitations. First, unlike experimental studies, the type of PWL or frequency of exposure cannot be controlled. However, this study is based on large-scale nationally representative data with more than 40,000 adolescents at about six months after the introduction of PWLs. Unlike a well-controlled experimental environment, our findings are meaningful because we evaluated the effects under real and complex scenarios, such as the wear-out effect over time caused by long-term exposure. Second, this study used the secondary data of a cross-sectional design survey measuring behavioral intention, not actual behavior (non-smokers’ intention not to start smoking and smokers’ intention to quit). Although it was not possible to evaluate the long-term causal effect of PWLs according to harm perception, it is also meaningful to evaluate intention because it is a key predictor of behavior in both the theory of planned behavior (one of the most used health behavior models) [25], and the tobacco warning model (model explaining the mechanism of PWL) [10]. The long-term harmful effects of smoking evoked by PWLs need to be confirmed through further longitudinal studies. Third, the result should be interpreted with caution since harm perception of smoking may have been sensitized for other reasons (i.e., family history of cancer, etc.), not by PWL. However, this possibility of sensitization by other factors can be minimized because the questionnaire directly asked whether the perception of harm induced by PWL.

Despite these limitations, this study is insightful as it provides evidence to countries that wish to introduce or strengthen the use of PWLs in terms of their effect on the possibility of reducing smoking prevalence among adolescents. The PWL rotation cycle in Korea is 24 months, and the PWL used in this survey was already updated once, and is about to be updated a second time, but the provisions (“That the warning pictures shall be based on facts without being overly obnoxious.”) still remain. Therefore, evidence from these studies can strengthen tobacco control policies related to the packaging of tobacco products. 

## 5. Conclusions

Harm perception evoked by PWLs predicts a higher non-smoking intention among adolescents. In particular, in the case of perceiving the harmfulness of smoking after exposure to PWLs, non-smoking intention was formed in about 45% of daily smokers who had no experience in trying to quit in the last 12 months. Therefore, in introducing PWLs, it is important to select those that can arouse sufficient awareness to increase their effectiveness. 

## Figures and Tables

**Table 1:** General characteristics of study population and warning reaction to pictorial warning labels.

Characteristics	All (Weighted % ^a^)	Warning Reactions	
		Harm Perception of Smoking	Non-Smoking Intention
Respondents (Unweighted N)	43,231		
**Overall**		83.9	83.1
**Harm perception of smoking**			
Yes	83.9	-	93.4
No	16.1	-	29.5
**Sex**			
Male	50.0	81.0	79.1
Female	50.0	86.8	87.1
**Grades**			
7th	14.9	94.3	93.7
8th	15.4	89.2	88.6
9th	15.0	87.0	86.2
10th	17.3	82.1	82.0
11th	18.8	78.7	77.8
12th	18.7	75.4	73.8
**Location**			
Metropolitan city	43.5	84.4	83.6
Small- or medium-sized city	50.5	83.5	82.7
Province	6.0	83.0	82.4
**Perceived household economic status**			
High	39.9	84.6	83.8
Middle	45.5	84.4	83.9
Low	14.6	80.3	78.5
**Perceived academic performance**			
High	40.3	85.0	85.2
Middle	28.4	85.1	85.0
Low	31.3	81.3	78.6
**Participation in smoking prevention education (last 12 months)**			
Yes	76.7	85.3	84.5
No	23.3	79.0	78.4
**Exposure to anti-tobacco message (last 12 months)**			
Yes	87.8	85.3	84.4
No	12.2	73.2	73.4
**Smoking status**			
Never smoker	84.1	88.0	89.0
Former smoker	8.0	74.8	70.7
Infrequent smoker (1~19 days/month) ^1)^	3.3	56.0	42.5
Frequent smoker (20~29 days/month) ^1)^	0.9	48.1	30.2
Daily smoker ^1)^	3.8	42.7	24.3

Data are presented as weighted percentage. a Rows may not be added up to 100% due to rounding. ^1)^ Current smokers.

**Table 2:** Evaluation of warning reaction attributed to the exposure to pictorial warning label.

Characteristics (N = 43,231)	Harm Perception of Smoking		Non-Smoking Intention		
	Unadjusted OR (95% CI)	Adjusted OR (95% CI)	Unadjusted OR (95% CI)	Model 1 Adjusted OR ^2)^ (95% CI)	Model 2 Adjusted OR ^3)^ (95% CI)
**Harm perception of smoking**					
Yes	-	-	33.87 (31.75–36.14) *	-	28.38 (26.37–30.53) *
No	-	-	Ref	-	Ref
**Sex**					
Male	Ref	Ref	Ref	Ref	Ref
Female	1.54 (1.44–1.64) *	1.13 (1.06–1.20) *	1.78 (1.66–1.90) *	1.18 (1.11–1.25) *	1.15 (1.06–1.23) *
**Grades**					
7th	5.42 (4.80–6.13) *	3.50 (3.08–3.97) *	5.32 (4.72–5.99) *	2.88 (2.55–3.26) *	1.73 (1.50–1.99) *
8th	2.71 (2.46–2.99) *	1.93 (1.74–2.13) *	2.76 (2.50–3.04) *	1.77 (1.60–1.95) *	1.33 (1.18–1.50) *
9th	2.19 (1.99–2.42) *	1.71 (1.55–1.89) *	2.22 (2.02–2.45) *	1.63 (1.48–1.80) *	1.30 (1.15–1.47) *
10th	1.50 (1.38–1.63) *	1.30 (1.19–1.41) *	1.62 (1.49–1.76) *	1.37 (1.25–1.50) *	1.26 (1.13–1.42) *
11th	1.21 (1.11–1.31) *	1.12 (1.02–1.22) *	1.25 (1.16–1.34) *	1.16 (1.07–1.26) *	1.12 (1.02–1.23) *
12th	Ref	Ref	Ref	Ref	Ref
**Location**					
Metropolitan city	1.10 (0.94–1.30)	0.95 (0.82–1.10)	1.09 (0.93–1.27)	0.88 (0.75–1.03)	0.86 (0.72–1.02)
Small- or medium-sized city	1.03 (0.88–1.22)	0.92 (0.80–1.07)	1.03 (0.88–1.20)	0.88 0.75–1.03)	0.88 (0.75–1.04)
Rural	Ref	Ref	Ref	Ref	Ref
**Perceived household economic status**					
High	Ref	Ref	Ref	Ref	Ref
Middle	0.98 (0.93–1.04)	1.00 (0.94–1.07)	1.01 (0.95–1.06)	1.02 (0.96–1.09)	1.02 (0.95–1.10)
Low	0.74 (0.69–0.80) *	0.93 (0.86–1.02)	0.71 (0.66–0.76) *	0.93 (0.85–1.01)	0.94 (0.85–1.04)
**Perceived academic performance**					
High	Ref	Ref	Ref	Ref	Ref
Middle	1.00 (0.94–1.08)	1.17 (1.09–1.26) *	0.98 (0.92–1.05)	1.16 (1.08–1.25) *	1.09 (1.00–1.19)
Low	0.76 (0.72–0.81) *	1.15 (1.07–1.23) *	0.64 (0.60–0.68) *	1.01 (0.94–1.09)	0.90 (0.83–0.98) *
**Participation in smoking prevention education (last 12 months)**					
Yes	1.55 (1.46–1.64) *	1.30 (1.22–1.39) *	1.51 (1.42–1.60) *	1.32 (1.24–1.41) *	1.21 (1.11–1.31) *
No	Ref	Ref	Ref	Ref	Ref
**Exposure to anti-tobacco message** **(last 12 months)**					
Yes	2.13 (2.00–2.27) *	1.63 (1.52–1.75) *	1.96 (1.83–2.10) *	1.43 (1.32–1.54) *	1.10 (0.99–1.21)
No	Ref	Ref	Ref	Ref	Ref
**Smoking status**					
Never smoker	9.87 (8.88–10.98) *	7.10 (6.33–7.96) *	25.28 (22.55–28.35) *	18.43 (16.34–20.79) *	15.80 (13.64–18.30) *
Former smoker	3.99 (3.51–4.52) *	3.60 (3.15–4.10) *	7.51 (6.56–8.60) *	6.80 (5.91–7.82) *	6.11 (5.11–7.31) *
Infrequent smoker (1~19 days/month) ^1)^	1.71 (1.47–1.99) *	1.47 (1.26–1.72) *	2.31 (1.96–2.71) *	1.99 (1.69–2.34)	1.96 (1.59–2.42) *
Frequent smoker (20~29 days/month) ^1)^	1.25 (0.99–1.57)	1.13 (0.89–1.42)	1.35 (1.06–1.73) *	1.23 (0.96–1.58)	1.20 (0.87–1.64)
Daily smoker ^1)^	Ref	Ref	Ref	Ref	Ref

* denoted significant associations (*p* < 0.05). ^1)^ Current smoker. ^2)^ Adjusted for sex, grade, location, perceived household economic status, perceived academic performance, participation in smoking prevention education (last 12 months), exposure to anti-tobacco message (last 12 months), and smoking status. ^3)^ Additional adjusted for harm perception of smoking to Model 1.

**Table 3:** Adjusted odds ratios for non-smoking intention caused by the exposure to pictorial warning label according to harm perception of smoking and smoking status.

Harm Perception of Smoking × Smoking Status		Non-Smoking Intention	
		Weighted %	Adjusted OR ^1)^ (95% CI)
No	Daily smoker ^2)^	3.7	Ref
	Frequent smoker (20~29 days/month) ^2)^	7.3	1.99 (1.10–3.59) *
	Infrequent smoker (1~19 days/month) ^2)^	12.3	3.37 (2.25–5.04) *
	Former smoker	21.0	6.50 (4.50–9.41) *
	Never smoker	40.1	15.24 (10.93–21.25) *
Yes	Daily smoker ^2)^	52.0	28.45 (19.95–40.59) *
	Frequent smoker (20~29 days/month) ^2)^	54.9	30.17 (19.68–46.26) *
	Infrequent smoker (1~9 days/month) ^2)^	66.2	47.07 (32.35–68.49) *
	Former smoker	87.4	169.16 (116.99–244.60) *
	Never smoker	95.7	466.11 (331.75–654.88) *
P for trend			<0.001

* denoted significant associations (*p* < 0.05). Those who had experience of exposure to pictorial warning label during the last 30 days (N = 43,231) were analyzed^. 1)^ Adjusted for sex, grade, location, perceived household economic status, perceived academic performance, participation in smoking prevention education (last 12 months), and exposure to anti-tobacco message (last 12 months). ^2)^ Current smoker.

**Table 4:** Adjusted odds ratios for non-smoking intention caused by the exposure to pictorial warning label according to harm perception and smoking frequency stratified by experience of trying to quit smoking among current smokers (N = 3226).

Harm Perception of Smoking × Smoking Frequency			Stratification by Experience of Trying to Quit Smoking during the Last 12 Months			
			No (N = 903)		Yes (N = 2323)	
			Weighted %	Adjusted OR ^1)^ (95% CI)	Weighted %	Adjusted OR ^1)^ (95% CI)
No	Daily smoker ^2)^		1.7	Ref	4.7	Ref
	Frequent smoker (20~29 days/month) ^2)^		6.9	4.12 (1.12–15.18) *	7.5	1.58 (0.81–3.07)
	Infrequent smoker (1~19 days/month) ^2)^		9.1	5.66 (2.47–13.00) *	13.6	3.21 (2.00–5.17) *
Yes	Daily smoker ^2)^		45.1	50.50 (23.59–108.13) *	54.2	24.44 (16.09–37.11) *
	Frequent smoker (20~29 days/month) ^2)^		43.6	52.75 (18.95–146.89) *	57.1	27.66 (17.05–44.89) *
	Infrequent smoker (1~19 days/month) ^2)^		58.8	90.70 (42.19–195.00) *	69.0	46.19 (29.82–71.56) *
P for trend			<0.001		<0.001	

* denoted significant associations (*p* < 0.05). ^1)^ Adjusted for sex, grade, location, perceived household economic status, perceived academic performance, participation in smoking prevention education (last 12 months), and exposure to anti-tobacco message (last 12 months). ^2)^ Current smoker.

## Data Availability

After approval of use, publicly available datasets were analyzed in this study. This data can be found here http://www.kdca.go.kr/yhs/.
